# KIF26B-AS1 Regulates TLR4 and Activates the TLR4 Signaling Pathway to Promote Malignant Progression of Laryngeal Cancer

**DOI:** 10.4014/jmb.2203.03037

**Published:** 2022-07-08

**Authors:** Li Li, Jiahui Han, Shujia Zhang, Chunguang Dong, Xiang Xiao

**Affiliations:** Department of Otolaryngology, Head and Neck Surgery, The First People’s Hospital of Lianyungang City, No.182, Tongguan Road, Haizhou District, Lianyungang City, Jiangsu Province 222100, P.R. China

**Keywords:** KIF26B-AS1, FUS, TLR4, TLR4 signaling pathway, laryngeal cancer

## Abstract

Laryngeal cancer is one of the highest incidence, most prevalently diagnosed head and neck cancers, making it critically necessary to probe effective targets for laryngeal cancer treatment. Here, real-time quantitative reverse transcription PCR (qRT-PCR) and western blot analysis were used to detect gene expression levels in laryngeal cancer cell lines. Fluorescence in situ hybridization (FISH) and subcellular fractionation assays were used to detect the subcellular location. Functional assays encompassing Cell Counting Kit-8 (CCK-8), 5-ethynyl-2’-deoxyuridine (EdU), transwell and wound healing assays were performed to examine the effects of target genes on cell proliferation and migration in laryngeal cancer. The in vivo effects were proved by animal experiments. RNA-binding protein immunoprecipitation (RIP), RNA pulldown and luciferase reporter assays were used to investigate the underlying regulatory mechanisms. The results showed that KIF26B antisense RNA 1 (KIF26B-AS1) propels cell proliferation and migration in laryngeal cancer and regulates the toll-like receptor 4 (TLR4) signaling pathway. KIF26B-AS1 also recruits FUS to stabilize TLR4 mRNA, consequently activating the TLR4 signaling pathway. Furthermore, KIF26B-AS1 plays an oncogenic role in laryngeal cancer via upregulating TLR4 expression as well as the FUS/TLR4 pathway axis, findings which offer novel insight for targeted therapies in the treatment of laryngeal cancer patients.

## Introduction

Laryngeal cancer is the third most prevalent head and neck cancer across the globe [1]. Epidemiological studies revealed that smoking and alcohol consumption are likely to increase the risk of laryngeal cancer [2, 3]. The treatment options for laryngeal cancer vary at different stages of disease: the treatment modalities for patients with T1-2 laryngeal cancer are radiotherapy, transoral laser microsurgery or open partial laryngectomy, and total laryngectomy alone or accompanied with radiotherapy and chemotherapy for those with locally advanced laryngeal cancer [4-6]. Though great progress has been achieved in clinical trials and therapies, there remain various side effects in terms of these therapeutic approaches for laryngeal cancer [7, 8]. Thus, it is of great importance to delve into novel targets for developing effective therapies against laryngeal cancer.

Long non-coding RNAs (lncRNAs) have been identified as non-coding transcripts characterized by over 200 nucleotides [9, 10]. Multiple lines of evidence support that dysregulated lncRNAs act as crucial regulators in various cancers [11-13], and laryngeal cancer is no exception [14-16]. For example, laryngeal cancer tissues exhibit a high expression level of SNHG1, which facilitates cell proliferation via inhibiting the Notch1 signaling pathway [17]; TUG1, which is highly expressed in tumor tissues, plasma samples of cancer patients, and tumor cells, facilitates the progression of laryngocarcinoma via activating the RhoA/ROCK/MMPs pathway [18]; AFAP1-AS1 is upregulated in laryngeal carcinoma specimens and propels cell stemness and chemoresistance in laryngeal carcinoma via miR-320a/RBPJ axis [19]; PCAT1, upregulated in laryngeal cancer tissues, enhances cell migration and invasion in laryngeal malignancy via sequestering miR-210-3p [20]. To ensure innovation in our study, we explored a novel dysregulated lncRNA whose role remains unclear in laryngeal cancer, namely KIF26B-AS1, and probed into its mechanism, which underlies laryngeal cancer.

RNA binding protein (RBP) FUS is related to the metabolism of RNA at various stages [21]. As indicated by literature review, FUS acts as an important regulator in multiple diseases. For instance, FUS modulates circHIF1A, which can propel the development of triple-negative breast cancer via targeting NFIB [22]; circSPARC propels p-STAT3 nuclear translocation by recruiting FUS in colorectal cancer (CRC) [23]; circRHOBTB3, which is transcriptionally activated by FUS, stabilizes PTBP1 mRNA to hamper metastasis of CRC [24]; and miR-4319 impairs the processes of thyroid cancer through regulating SMURF1, which is stabilized by FUS [25]. In addition, it has been reported that circ_0006232 modulates the stability of EZH2 mediated by FUS, thereby facilitating the processes of laryngeal squamous cell cancer, a common subtype of laryngeal malignancy [26]. However, the correlation of FUS with KIF26B-AS1 has never been reported in laryngeal cancer.

Toll-like receptors (TLRs) are molecules featuring evolutionary conservatism and a capacity to regulate innate and adaptive immunity. The role of the TLR family has been reported in head and neck cancers. TLR-9, TLR-5, TLR-4, TLR-3, TLR-2 and TLR-1 are linked to some clinical parameters of patients afflicted with head and neck squamous cell carcinoma (HNSCC) [27]. Nevertheless, the relationship between TLRs and laryngeal cancer has rarely been reported.

In the present study, we investigated the aberrant expression of KIF26B-AS1 in laryngeal cancer cell lines and explored its biological role in laryngeal cancer through a series of functional assays. Furthermore, mechanism investigation into how KIF26B-AS1 regulates laryngeal cancer progression via TLR signaling pathway and FUS was also performed. Clarifying the molecular mechanism underlying laryngocarcinoma may contribute to the advance of its targeted therapies.

## Materials and Methods

### Cell Culture

Human laryngeal carcinoma cell lines (TU686, H4E, AMC-HN-8) and human normal bronchial epithelial cell line BEAS-2B were commercially acquired from ATCC (USA) and incubated in RPMI 1640 medium (CD-02168-Gibco, USA) containing 10% fetal bovine serum (FBS, 10270-106, Gibco) and 100 U/ml penicillin-streptomycin solutions. The incubator was humidified with 5% CO_2_ at 37°C.

### Vector Construction and Cell Transfection

The full length of KIF26B-AS1/FUS/TLR4 was inserted into the pcDNA3.1 vector to build their overexpression vectors with the empty pcDNA3.1 vector as the negative control. Short hairpin RNAs (shRNAs) of KIF26B-AS1/FUS were utilized to for interference of RNAs with sh-NC as negative control. Then, 48 h subsequent to transfection of lentivirus-containing shRNAs, the cells were selected by puromycin for 14 days. The cell lines stably transfected with vectors were established, with transfection efficiency being validated. All the shRNAs and lentiviral vector GV358 were commercially attained from RiboBio (China). Lipofectamine 2000 (XFSJ16444, Gibco) was applied for plasmid transfection. The sequences of shRNAs were provided as displayed in Table S1.

### Bioinformatics Analysis

RBPs combining with KIF26B-AS1 were forecasted by starBase v2.0 (http://starbase.sysu.edu.cn/) using published CLIP-seq from GEO [28]. Kyoto Encyclopedia of Genes and Genomes (KEGG, https://www.kegg.jp/) is a database resource for interpreting high-throughput data [29]. Gene Set Enrichment Analysis (GSEA, http://www.gsea-msigdb.org/gsea/index.jsp) can identify the differences of specific sets of genes between two biological states [30]. With certain parameters (numbers of permutations: 2000), GSEA v7.5.1 was applied for the enrichment analysis of KEGG v7.5.1 datasets from c2.cp.kegg.v7.5.1.symbols. Hum-mPLoc 2.0 (http://www.csbio.sjtu.edu.cn/bioinf/Cell-PLoc-2/) was utilized for the prediction of subcellular localization of FUS [31]. lncLocator 1.0 (http://www.csbio.sjtu.edu.cn/bioinf/lncLocator/) acted as a subcellular localization predictor to analyze the localization of KIF26B-AS1 [32].

### Animal Study

BALB/c nude mice (male, 5~6 weeks old, 20 ± 2 g) were commercially attained from Shanghai Laboratory Animal Co., Ltd., China). Approval was obtained for all the animal studies from The First People’s Hospital of Lianyungang City. Nude mice were subgrouped into three groups with three mice in each group, followed by being subcutaneously injected with H4E cells stably transfected with sh-NC, sh-KIF26B-AS1-1 or sh-KIF26B-AS1-2. Xenograft tumor volume was measured and recorded every three days after one week post- injection. Then, 28 days later, the nude mice were sacrificed prior to the tumor excision. The tumor tissue, after the removal, was weighed and subjected to immunohistochemistry (IHC) analysis for the detection of cell proliferation marker Ki67.

### Real-Time Quantitative Reverse Transcription PCR (qRT-PCR)

Total RNAs were isolated by Trizol (abs60154, China), followed by conversion into cDNA with reverse transcription. Then, qRT-PCR was carried out with the application of ChamQ Universal SYBR qPCR Master Mix (Q711-02, Vazyme Biotech, China). The relative RNA expression was counted on the basis of 2^-ΔΔCt^. GAPDH was treated as internal reference.

### Western Blot

Cells were lysed in RIPA lysis buffer (RIPA20110527, TBD, China) and treated with a ProteoPrep Total Extraction Sample Kit (PROTTOT-1KT, Sigma-Aldrich, USA) to extract total proteins. The Bradford Protein Assay (PC0010, Solarbio, China) was applied to analyze protein concentration. Subsequently, the proteins were separated on SDS-PAGE (P1200, Solarbio), followed by being transferred onto PVDF membranes. After that, membranes were blocked with 5% non-fat milk. Then, the primary antibodies were used for incubation with blots. After being washed, blots were then incubated with secondary antibodies. ECL detection was utilized to visualize the blots. The antibodies adopted in this experiment included anti-Vimentin (ab92547), anti-E-cadherin (ab40772), anti-N-cadherin (ab76011), anti-β-actin (ab8226), anti-TLS/FUS (ab243880), anti-MYD88 (ab219413), anti-TLR4 (ab13556), anti-IRAK1 (ab180747) and anti-TRAF6 (ab137452). All the antibodies were commercially acquired from Abcam (UK).

### Cell Proliferation Assays

Cell Counting Kit-8 (CCK-8) and 5-ethynyl-2’-deoxyuridine (EdU) assays were implemented to analyze H4E and AMC-HN-8 cell proliferation. CCK-8 reagent (MedChemExpress, USA) was applied for the implementation of CCK-8 assays. Transfected cells were incubated in 96-well plates. The incubation of cells continued 24, 48 and 72 h, and then cells were subjected to incubation with CCK-8 solution for another 4 h. The absorbance at 450 nm was detected using a microplate reader. For the EdU assays, transfected cells were seeded in 12-well plates, and EdU reagent (RiboBio) was applied to stain cells in the proliferation process. DAPI was utilized to color cell nuclei. EdU-labeled cells were counted via a fluorescence microscope.

### Transwell Assay

The Transwell assay was applied to uncover the migratory ability of H4E and AMC-HN-8 cells. Cells were incubated in the upper layer of the Transwell chamber which was filled with serum-free medium. Additionally, complete medium was added to the lower layer. Then, 24 h later, cells in the upper chamber were abraded slightly with a cotton swab. Following that, cells located on the bottom side of the membrane were subjected to fixation utilizing methanol solution and stained with crystal violet. By using an optical microscope, migrated cells were imaged and analyzed.

### Wound Healing Assay

When cell confluence reached 90%, cells in 24-well plates were scratched by a pipette tip to create a straight wound. After PBS washing, the wound images were taken immediately and recorded as the width of 0 h. Then, 24 h later, the wound was imaged again. Bio-repeats of the assay were implemented in triplicate.

### Fluorescence In Situ Hybridization (FISH)

Cells inoculated in 12-well plates were subjected to treatment with 4% paraformaldehyde for fixation and 0.5%Triton X-100 for cell permeabilization. After the treatment of proteinase K solution and glycine buffer, cells were subjected to incubation in hybridization solution with probes at 37°C overnight. DAPI was utilized to dye the cell nuclei. All images of the location of KIF26B-AS1 and FUS were acquired by a Zeiss LSM 880 confocal microscope. The sequences of probes were added in Table S2.

### Subcellular Fractionation

Subcellular fractionation assay was performed as described previously [33]. A Nuclear/Cytosol Fractionation Kit (K266-25, Amyjet Scientific, China) was employed to separate the nucleus and cytoplasm of laryngeal carcinoma cells. The total RNA isolated from the nucleus and cytoplasm was subjected to qRT-PCR analysis. β-Actin and U6 acted as internal controls for cytoplasm and nucleus respectively.

### RNA-Binding Protein Immunoprecipitation (RIP)

Three groups were set as Input, IgG and the target protein group. Five micrograms of antibodies of the target protein and 5 μg anti-IgG were incubated with 50 μg Protein A/G Agarose magnetic beads at 4°C overnight. By treating 10% lysate as the Input group, 100 μl cell lysis buffer was added into IgG and target protein groups respectively and cultured with the antibody-magnetic beads complex at 4°C for another whole night. After non-specific washing and RNA purification, RNA enrichment was disclosed by qRT-PCR analysis.

### Luciferase Reporter Assay

The pmirGLO-TLR4 3’UTR plasmid was constructed by inserting TLR4 3’UTR into pmirGLO vector. After that, pmirGLO-TLR4 3’UTR were co-transfected into H4E cells with pcDNA3.1-KIF26B-AS1/pcDNA3.1-FUS. The luciferase activity was detected with Renilla luciferase as an endogenous reference. The pGL3-TLR4 promoter was built by inserting full-length sequences of TLR4 promoter (2,000 bp upstream of TSS) into pGL3 vector with the empty pGL3 vector used as control. The luciferase activity was measured 36 h after the co-transfection of pGL3-TLR4 promoter and pcDNA3.1-KIF26B-AS1/pcDNA3.1-FUS. The luciferase activity was unveiled with a Dual Luciferase Reporter Gene Assay Kit (RG027, Beyotime, China) following the manufacturer’s instructions.

### RNA Pulldown Assay

Biotinylated TLR4 3’UTR or KIF26B-AS1/TLR4 sense were incubated with Structure Buffer (1 ml lysis buffer; 1% Triton X-100, 10 mM HEPES pH 7.0, 200 mM NaCl, 10 mM MgCl2, 1 mM DTT with protease inhibitors and RNase inhibitor) to form a secondary structure. Then, streptavidin magnetic beads and bio-TLR4 3’UTR or bio-KIF26B-AS1/TLR4 sense were cocultured in cell lysis buffer at 4°C overnight. For RNA-RNA pull-down, after a brief centrifugation, RNAs were extracted from the complex and conducted by qRT-PCR analysis. For RNA-protein pull-down, after a brief centrifugation, the complex was treated with RIP Wash Buffer and 5×SDS Loading Buffer and then subjected to western blot analysis.

### mRNA Stability Assay

The mRNA stability assay was applied in H4E and AMC-HN-8 cells subsequent to treatment of 50 mM α-amanitin. TLR4 transcription was probed by qRT-PCR analysis at 0, 6, 12, 18, and 24 h respectively.

### Statistical Analysis

Statistical analyses of all the experiments were implemented with triplicate biological repeats. The data were exhibited as mean ± SD, with SPSS 22.0 utilized for data analysis. For multiple comparisons, Student’s *t*-test and one-way/two-way analysis of variance (ANOVA) were used. *p* < 0.05 was indicative of the threshold for statistical significance.

## Results

### KIF26B-AS1 Promotes the Proliferative and Migratory Capacities of Laryngeal Cancer Cells In Vitro

**Fig. 1:**
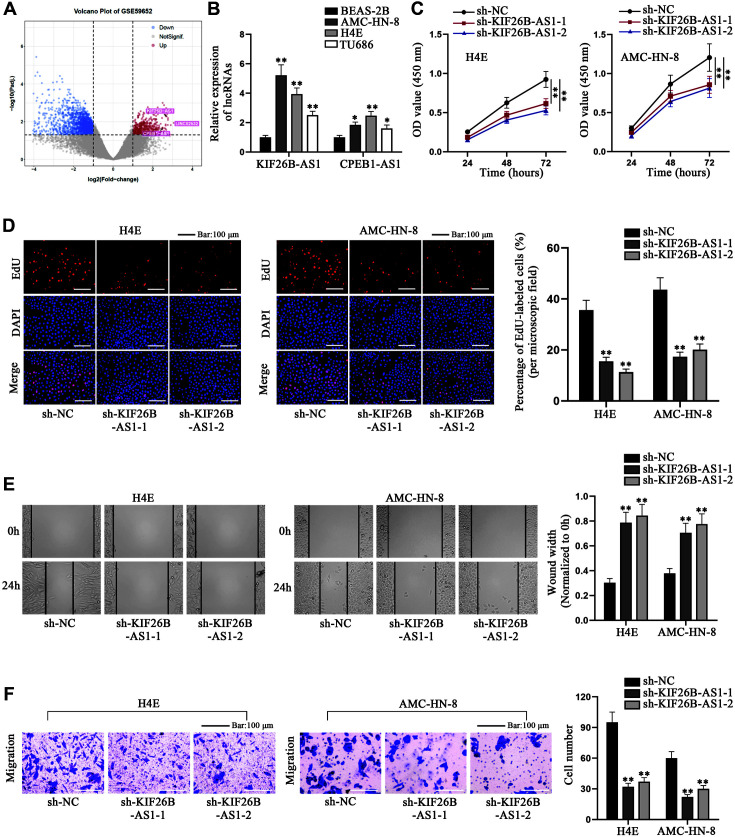
KIF26B-AS1 promotes the proliferative and migratory abilities of laryngeal cancer cells in vitro. (**A**) GEO database was retrieved to probe the differentially expressed RNAs in laryngeal cancer tissues. (**B**) The expressions of KIF26B-AS1 and CPEB1-AS1 were detected by qRT-PCR in BEAS-2B, AMC-HN-8, H4E and TU686 cells. (**C, D**) CCK-8 and EdU assays detected the proliferation of H4E and AMC-HN-8 cells after the transfection of sh-KIF26B-AS1-1/2. (**E, F**) Wound healing and Transwell assays were conducted to unveil the migration of H4E and AMC-HN-8 cells subsequent to the inhibition of KIF26B-AS1. One-way ANOVA was applied to compare differences. * *p* < 0.05, **p < 0.01.

We used GEO database to explore the lncRNAs differentially expressed in laryngeal cancer tissues relative to that in adjacent tissues. In accordance with GSE59652 dataset, we screened out 3 lncRNAs, LINC02532, KIF26B-AS1 and CPEB1-AS1 (Fig. 1A). Based on a previous report, LINC02532 is overexpressed in gastric cancer [34]. Nevertheless, the role of KIF26B-AS1 and CPEB1-AS1 in laryngeal malignancy still remained unclear; hence we explored these two lncRNAs in laryngeal carcinoma cells. The expressions of KIF26B-AS1 and CPEB1-AS1 in human normal bronchial epithelial cell line (BEAS-2B) and laryngeal cancer cell lines (AMC-HN-8, H4E, TU686) were uncovered by qRT-PCR. The results showed that KIF26B-AS1 was significantly overexpressed in the laryngeal cancer cell lines relative to BEAS-2B, indicating the association of KIF26B-AS1 with laryngeal cancer (Fig. 1B). We selected KIF26B-AS1 as the focus of study. Moreover, due to the relatively higher expression of KIF26B-AS1, AMC-HN-8 and H4E were chosen for the follow-up experiments. We implemented qRT-PCR to detect the efficiency of sh-KIF26B-AS1-1/2/3 or pcDNA3.1-KIF26B-AS1 (Figs. S1A and S1B). Because of the higher efficiency, sh-KIF26B-AS1-1 and sh-KIF26B-AS1-2 were chosen for experiments. Next, we performed loss-of-function experiments using sh-KIF26B-AS1-1/2. It was disclosed by CCK-8 and EdU assays that AMC-HN-8 and H4E cell proliferation was inhibited after the inhibition of KIF26B-AS1, as evidenced by the decrease in the OD value and EdU-labeled cells (Figs. 1C and 1D). It was manifested by wound healing and Transwell assays that KIF26B-AS1 ablation decreased the healing speed and the number of migrated cells, suggesting that KIF26B-AS1 interference hindered the migration of laryngeal cancer cells (Figs. 1E and 1F). To sum up, KIF26B-AS1 promotes the proliferative and migratory abilities of laryngeal cancer cells in vitro.

### KIF26B-AS1 Positively Regulates TLR4 Signaling Pathway in Laryngeal Carcinoma Cells

**Fig. 2:**
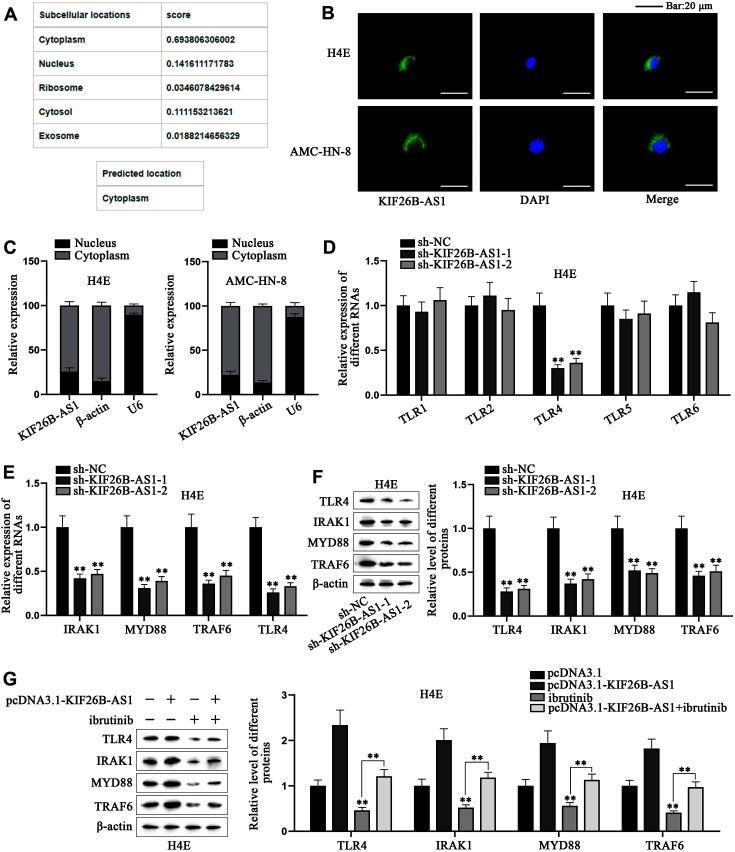
KIF26B-AS1 positively regulates TLR4 signaling pathway in laryngeal cancer cells. (**A**) The subcellular distribution of KIF26B-AS1 was predicted via using lncLocator (http://www.csbio.sjtu.edu.cn/bioinf/lncLocator/). (**B, C**) FISH (with β-actin and U6 as internal control) and nucleus-cytoplasm fractionation assays detected the subcellular location of KIF26B-AS1 in H4E and AMC-HN-8 cells. (**D**) The expressions of TLR1, TLR2, TLR4, TLR5 and TLR6 in H4E cells were detected by qRT-PCR after the ablation of KIF26B-AS1. (**E, F**) The levels of TLR4, IRAK1, MYD88 and TRAF6 in H4E cells were investigated by qRT-PCR and western blot after the knockdown of KIF26B-AS1. (**G**) The levels of TLR4, IRAK1, MYD88 and TRAF6 in H4E cells were probed by western blot after the overexpression of KIF26B-AS1 and the addition of ibrutinib. Student’s *t*-test and one-way/two-way ANOVA were adopted for comparing differences. **p < 0.01.

Next, we explored the modulatory mechanism of KIF26B-AS1 in laryngeal cancer cells. First, we detected the subcellular location of KIF26B-AS1 in laryngeal cancer cells. We forecasted the location of KIF26B-AS1 via lncLocator, and found that KIF26B-AS1 was predicted to be located in both cytoplasm and nucleus, but mainly in cytoplasm (Fig. 2A). We performed FISH and nucleus-cytoplasm fractionation assays to verify the prediction (Figs. 2B and 2C). KEGG predicted four pathways regulated by KIF26B-AS1, namely, extracellular matrix–receptor (ECM-receptor) combination, HEMATOPOIETIC/CELL/LINEAGE, SYSTEMIC/LUPUS/ERYTHEMATOSUS and TLR signaling pathways (Fig. S2A). A previous study has shown that the TLR signaling pathway plays a crucial role in HNSCC [35]. Afterwards, we used GSEA to predict the mRNAs linked to KIF26B-AS1. The results showed that the mRNAs of TLR signaling pathway were correlated with KIF26B-AS1 (Fig. S2B). Therefore, KIF26B-AS1 might activate TLR signaling pathway. For verification, we conducted qRT-PCR to investigate the influence of KIF26B-AS1 on the mRNA levels of TLR1, TLR2, TLR4, TLR5 and TLR6, the related proteins of TLR pathway [36]. The results showed that KIF26B-AS1 inhibition only significantly knocked down the expression of TLR4 mRNA in H4E cells (Fig. 2D). Hence, we studied the correlation between KIF26B-AS1 and TLR4 signaling. We conducted qRT-PCR to unveil the effect of KIF26B-AS1 on TLR4, IRAK1, MYD88 and TRAF6, the related proteins of TLR4 signaling pathway [37]. We found that KIF26B-AS1 ablation decreased the mRNA levels of these proteins in H4E cells, especially TLR4 (Fig. 2E). The results of western blot proved that KIF26B-AS1 depletion decreased TLR4, IRAK1, MYD88 and TRAF6 protein levels in H4E cells, especially TLR4 (Fig. 2F). We then performed western blot again and found that the overexpression of KIF26B-AS1 increased the expressions of TLR4, TRAF6, IRAK1 and MYD88, which was reversed by the addition of pathway inhibitor ibrutinib (Fig. 2G). The above results indicated that KIF26B-AS1 positively regulates TLR4 signaling pathway in laryngeal cancer cells.

### KIF26B-AS1 Recruits FUS to Stabilize TLR4 mRNA and Activate TLR4 Signaling Pathway

**Fig. 3:**
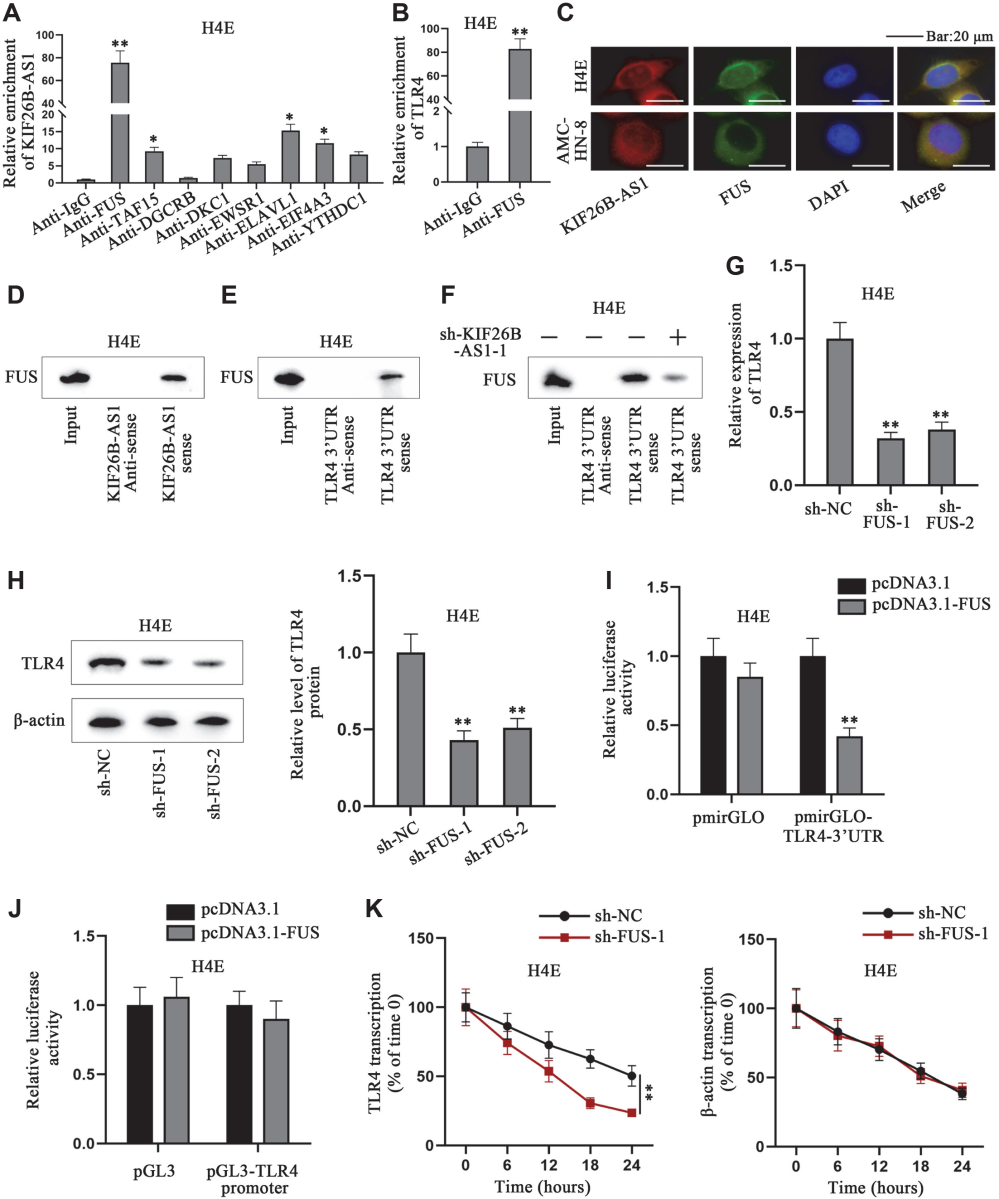
KIF26B-AS1 recruits FUS to stabilize TLR4 mRNA and activate TLR4 signaling pathway. (**A**) RIP assay was performed in H4E cells to investigate the enrichment of KIF26B-AS1 in Anti-IgG, Anti-FUS, Anti-TAF15, Anti-DGCRB, Anti-DKC1, Anti-EWSR1, Anti-ELAVL1, Anti-EIF4A3 and Anti-YTHDC1 groups. (**B**) RIP assay probed TLR4 enrichment in Anti-FUS group in H4E cells. (**C**) FISH detected the co-localization of KIF26B-AS1 and FUS in AMC-HN-8 and H4E cells. (**D**) RNA pulldown assay was carried out in H4E cells to detect FUS enrichment in KIF26B-AS1 sense or Anti-sense group. (**E**) RNA pulldown assay was implemented in H4E cells to disclose FUS enrichment in TLR4 sense or Anti-sense group. (**F**) RNA pulldown assay in H4E cells uncovered the effect of KIF26B-AS1 knockdown on FUS enrichment in TLR4 sense or Anti-sense group. (**G, H**) Western blot and qRT-PCR were performed in H4E cells to evaluate the level of TLR4 after the knockdown of FUS. (**I, J**) Luciferase reporter assays detected the relationship between FUS and TLR4 3’UTR/promoter in H4E cells. (**K**) The stability of TLR4 in H4E cells was unveiled by qRT-PCR after the depletion of FUS. Student’s *t*-test and one-way/two-way ANOVA were utilized for comparing differences. *p < 0.05, **p < 0.01.

According to the results of FISH shown in Fig. 2, we speculated that RBP might participate in the regulation of TLR4 signaling pathway with KIF26B-AS1. A previous study indicated that RBP plays a significant role in laryngeal cancer [38]. Therefore, we used starBase to screen out the potential RBPs of KIF26B-AS1, namely, FUS, TAF15, DGCRB, DKC1, EWSR1, ELAVL1, EIF4A3 and YTHDC1 (Fig. S3A). For verification, we conducted RIP in H4E cells and found that KIF26B-AS1 was most enriched in Anti-FUS group, proving their interaction (Fig. 3A). Furthermore, the interaction between FUS and TLR4 was predicted by starBase (Fig. S3B). RIP was conducted again to prove the interaction between FUS and TLR4 mRNA (Fig. 3B). Based on the above results, we selected FUS for follow-up assays. We used Hum-mPLoc 2.0 to predict that FUS was distributed in both nucleus and cytoplasm (Fig. S3C). We then conducted FISH for verification. The results showed that KIF26B-AS1 and FUS were co-localized in H4E and AMC-HN-8 cell cytoplasm (Fig. 3C). Subsequently, we performed RNA pulldown assays to prove the binding between KIF26B-AS1 and FUS, TLR4 mRNA and FUS (Figs. 3D and 3E). However, the level of FUS pulled down by TLR4 mRNA was inhibited by KIF26B-AS1 knockdown, showing that KIF26B-AS1 ablation suppressed the interaction between FUS and TLR4 mRNA (Fig. 3F). We implemented qRT-PCR to detect the efficiency of sh-FUS-1/2/3 or pcDNA3.1-FUS (Figs. S3D and S3E). Based on the results, sh-FUS-1 and sh-FUS-2 were selected owing to the high efficiency. It was indicated by western blot and qRT-PCR that after the inhibition of FUS, TLR4 was inhibited at the level of mRNA and protein in H4E cells (Figs. 3G and 3H). Luciferase reporter assays disclosed the relationship between FUS and TLR4 3’UTR or TLR4 promoter. As disclosed in Fig. 3I, luciferase activity of pmirGLO-TLR4-3’UTR was significantly reduced after co-transfection of pcDNA3.1-FUS compared with control group, indicating that TLR4 3’UTR stability is attenuated by FUS overexpression. As displayed in Fig. 3J, luciferase activity of pGL3-TLR4 promoter remained unchanged after FUS overexpression compared with control group, suggesting that FUS cannot influence the promoter activity of TLR4. After the treatment of α-amanitin (inhibitor of transcription), TLR4 expression level was uncovered by qRT-PCR in H4E cells every 6 h. The results showed that FUS could reinforce TLR4 mRNA stability (Fig. 3K). Taken together, KIF26B-AS1 recruits FUS to stabilize TLR4 mRNA and activate TLR4 signaling pathway.

### KIF26B-AS1 Regulates TLR4 Expression to Promote the Proliferative and Migratory Abilities of Laryngeal Carcinoma Cells

**Fig. 4:**
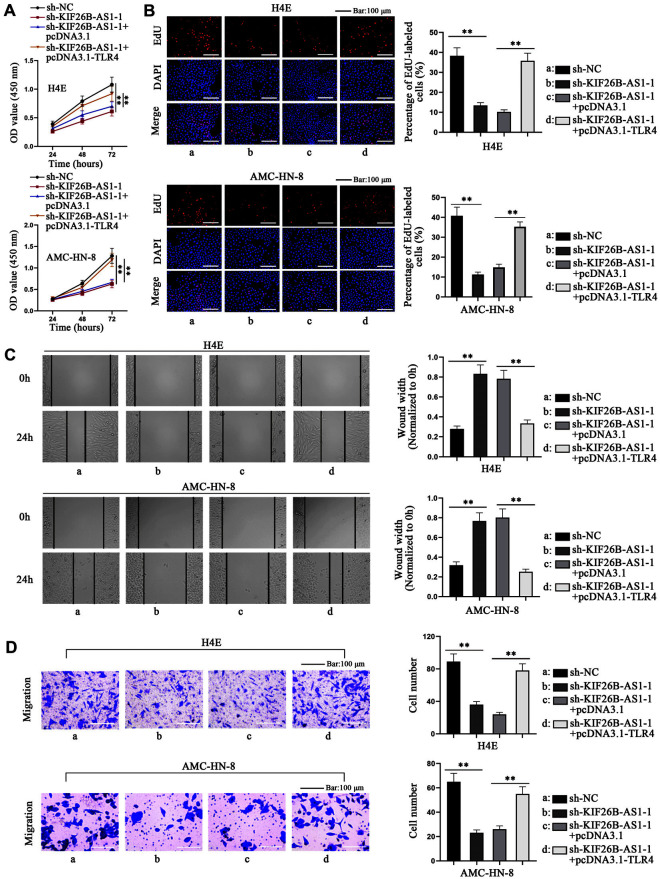
KIF26B-AS1 modulates TLR4 expression to promote the proliferative and migratory abilities of laryngeal carcinoma cells. (**A, B**) CCK-8 and EdU assay were conducted to detect the proliferation of H4E and AMC-HN- 8 cells subsequent to the transfection with sh-NC, sh-KIF26B-AS1-1, sh-KIF26B-AS1-1+pcDNA3.1 and sh-KIF26B-AS1- 1+pcDNA3.1-TLR4. (**C, D**) The migration of H4E and AMC-HN-8 cells was assessed by wound healing and Transwell assays after the transfection with sh-NC, sh-KIF26B-AS1-1, sh-KIF26B-AS1-1+pcDNA3.1 and sh-KIF26B-AS1-1+pcDNA3.1- TLR4. One-way ANOVA was used for comparing differences. **p < 0.01.

We then used the rescue experiments to verify whether KIF26B-AS1 could propel the proliferation and migration of laryngeal carcinoma cells by regulating the expression of TLR4. We carried out qRT-PCR to assess the efficiency of pcDNA3.1-TLR4 (Fig. S3F). Via functional experiments, we found that cell migration and proliferation impaired by KIF26B-AS1 ablation were reversed by the overexpression of TLR4 (Figs. 4A-4D). Taken together, KIF26B-AS1 regulates TLR4 expression to augment proliferation and migration of laryngeal cancer cells.

### KIF26B-AS1 Promotes Laryngeal Cancer Cell Progression In Vivo

**Fig. 5:**
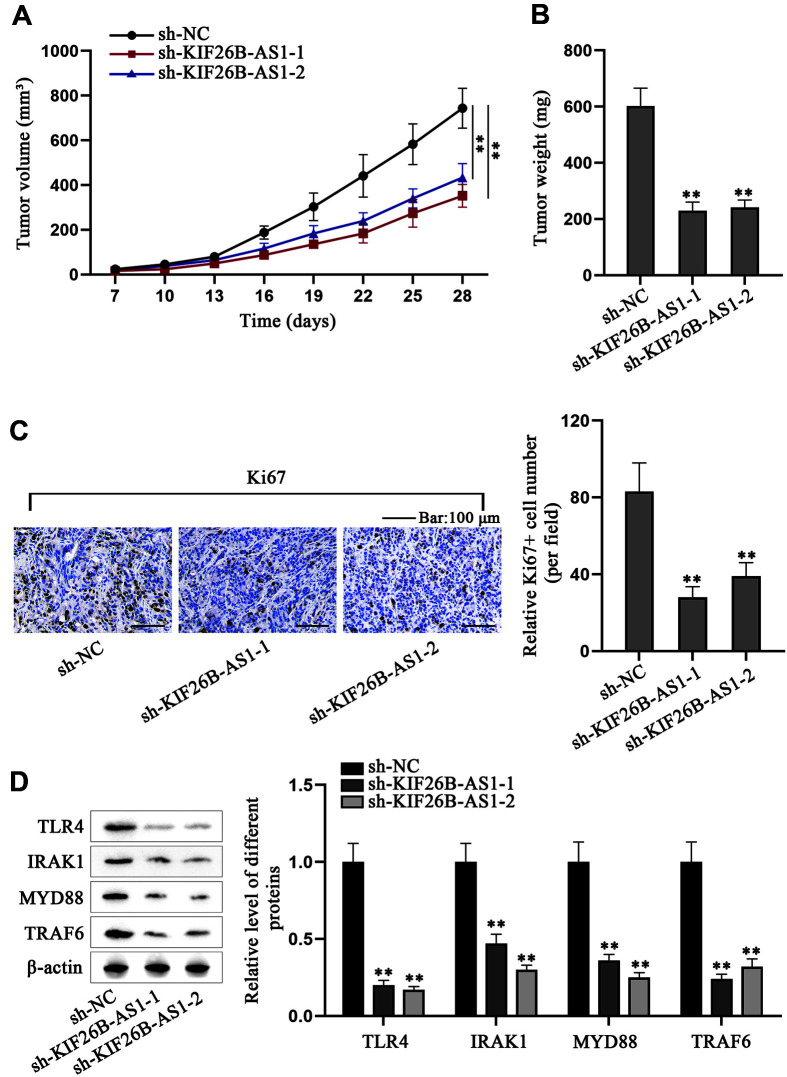
KIF26B-AS1 promotes laryngeal cancer cell progression in vivo. (**A**) 7 dpi, tumor volume was calculated every 3 days. (**B**) Tumor weight was analyzed 28 dpi. (**C**) IHC was used to measure Ki67 in tumor tissues. (**D**) Western blot detected TLR4, IRAK1, MYD88 and TRAF6 protein levels in tumor tissues after the knockdown of KIF26B-AS1. One-way ANOVA was utilized to compare differences. **p < 0.01.

We performed animal experiments to further prove the effects of KIF26B-AS1 on laryngeal cancer. The H4E cells, subsequent to transfection with sh-NC or sh-KIF26B-AS1-1, were injected subcutaneously into the nude mice for the experiments. We observed the tumor volume for 28 days post-injection (dpi). It was found that tumor growth in sh-KIF26B-AS1-1/2 group was inhibited (Fig. 5A). Then, 28 dpi, the tumors were resected from the sacrificed mice and measured, showing that tumor weight was decreased by KIF26B-AS1 knockdown (Fig. 5B). Subsequently, we performed IHC to detect Ki67 in tumor tissues and found that the Ki67-positive cell number in sh-KIF26B-AS1-1/2 group was lower than that in sh-NC group (Fig. 5C). Western blot analyses were then carried out to investigate the levels of TLR4 signaling pathway-related proteins in the tissues, with the results showing that KIF26B-AS1 inhibition decreased the levels of these proteins (Fig. 5D). Taken together, KIF26B-AS1 promotes laryngeal cancer cell progression in vivo.

## Discussion

Laryngeal cancer is one of the most frequent head and neck malignancies [39]. Despite the progress in clinical research and treatment, the serious side effects of current therapeutic methods undermine the overall survival of laryngeal cancer patients [7, 8]. Hence, it is urgent to probe into novel targets to develop effective therapies.

In recent years, lncRNAs, according to literature review, have come to serve as crucial regulators in various malignancies, including laryngeal cancer [14]. For instance, NKILA [40], MSC-AS1[41], lncRNA-ATB [42], RP11-159K7.2 [43], as well as LINC00152 [44] have been reported to modulate laryngeal cancer progression via different regulatory mechanisms. In our study, we used bioinformatics to screen the lncRNAs that were differentially expressed in laryngeal cancer tissues relative to the adjacent tissues. Following the results of qRT-PCR analysis, we selected KIF26B-AS1 as the focus of our study due to its aberrantly significant upregulation in laryngeal carcinoma cell lines. Afterwards, we performed a series of functional assays to evaluate the effects of KIF26B-AS1 on the biological functions of laryngeal cancer cells. Cell proliferation assays showed that KIF26B-AS1 ablation inhibited the proliferation of laryngeal cancer cells. Wound healing and Transwell migration assays disclosed that KIF26B-AS1 ablation hampered the migration of laryngeal cancer cells. Furthermore, animal experiments proved the effects of KIF26B-AS1 on laryngeal tumor growth in vivo. To sum up, KIF26B-AS1 promotes the proliferative and migratory abilities of laryngeal carcinoma cells in vitro and in vivo. With the help of bioinformatics, we found that KIF26B-AS1 might regulate TLR signaling pathway. FISH and nucleus-cytoplasm fractionation assays revealed that KIF26B-AS1 was mainly distributed in cytoplasm. Furthermore, through the detection of related genes, we found that KIF26B-AS1 was a positive regulator of TLR4 signaling pathway. TLR4 signaling pathway has been reported to play a crucial role in various cancers, including human oral squamous cell carcinoma [45], ovarian cancer [46, 47] and prostate cancer [48]. Next, we further probed into the underlying molecular mechanism of KIF26B-AS1 in regulating TLR4 signaling pathway in laryngeal malignancy.

Based on literature review, lncRNAs combine with RBP to regulate laryngeal cancer [49]. Also with the help of bioinformatics, we obtained several putative RBPs of KIF26B-AS1. RIP assays further determined the potential RBP which binds with KIF26B-AS1 in laryngeal cancer, namely FUS. We referred to previous studies and found that FUS participates in the regulation of various cancers. For instance, FUS, modulated by circRNA_0000285, has been found to facilitate the development of cervical cancer [50]; FUS combines with circ0005276 to propel cell proliferation and migration of prostate cancer via the activation of XIAP transcription [51]; FUS participates in the development of thyroid cancer through the stabilization of SMURF1 [25]; and LINC00205 recruits FUS to facilitate the progression of lung malignancy [52]. In our study, we researched the role of FUS in laryngeal cancer cells. We determined the co-localization of KIF26B-AS1 and FUS in cytoplasm of laryngeal carcinoma cells. Through mechanism experiments, we found that FUS could interact with KIF26B-AS1 and TLR4 3’UTR, and KIF26B-AS1 could inhibit the interaction between FUS and TLR4. FUS could stabilize TLR4 mRNA via mRNA stability assay. Afterwards, rescue experiments showed that KIF26B-AS1 regulated TLR4 signaling pathway via FUS.

In conclusion, the current study manifested that KIF26B-AS1 regulates TLR4 and activates the TLR4 pathway to promote the malignant progression of laryngeal cancer. In addition, our study demonstrated the underlying mechanisms of KIF26B-AS1 in laryngeal cancer, offering insight into targeted therapies for laryngeal malignancy. However, the present study lacks clinicopathological analysis, which will be included in further exploration to corroborate the clinical value of KIF26B-AS1 in laryngeal cancer. We will further probe into the correlation between KIF26B-AS1 expression level and the clinicopathological features of laryngeal cancer patients.

## Ethics Approval

Approval for all the animal studies was obtained from the Ethics Committee of First People’s Hospital of Lianyungang City.
